# Hydrogen sulfide attenuates chronic restrain stress-induced cognitive impairment by upreglulation of Sirt1 in hippocampus

**DOI:** 10.18632/oncotarget.22237

**Published:** 2017-11-01

**Authors:** Xiao-Na Li, Lei Chen, Bang Luo, Xiang Li, Chun-Yan Wang, Wei Zou, Ping Zhang, Yong You, Xiao-Qing Tang

**Affiliations:** ^1^ Institute of Neuroscience, Hunan Province Cooperative Innovation Center for Molecular Target New Drug Study, Medical College, University of South China, Hengyang 421001, Hunan, P. R. China; ^2^ Department of Physiology, Medical College, University of South China, Hengyang 421001, Hunan, P. R. China; ^3^ Department of Neurology, Nanhua Affiliated Hospital, University of South China, Hengyang 421001, Hunan, P. R. China; ^4^ Department of Neurology, The First Affiliated Hospital, University of South China, Hengyang 421001, Hunan, P. R. China; ^5^ Department of Anaesthesiology, The First Affiliated Hospital, University of South China, Hengyang 421001, Hunan, P. R. China; ^6^ Department of Pathophysiology, Medical College, University of South China, Hengyang 421001, Hunan, P. R. China

**Keywords:** cognitive impairment, chronic-restrain-stress, hydrogen sulfide, hippocampal damage, silence information regulator-1

## Abstract

Chronic restraint stress (CRS) has detrimental effects on cognitive function. Hydrogen sulfide (H_2_S), as a neuromodulator, regulates learning and memory. Hippocampus is a key structure in learning and memory. Sirt1 (silence signal regulating factor 1) plays an important role in modulating cognitive function. Therefore, our present work was to investigate whether H_2_S meliorates CRS-induced damage in hippocampus and impairment in cognition, and further to explore whether the underlying mechanism is via upreglulating Sirt1. In our present work, the behavior experiments [Y-maze test, Novel object recognition (NOR) test, Morris water maze (MWM) test] showed that sodium hydrosulfide (NaHS, a donor of H_2_S) blocked CRS-induced cognitive impairments in rats. NaHS inhibited CRS-induced hippocampal oxidative stress as evidenced by decrease in MDA level as well as increases in GSH content and SOD activity. NaHS rescued CRS-generated ER stress as evidenced by downregulations of CPR78, CHOP, and cleaved caspase-12. NaHS reduced CRS-exerted apoptosis as evidenced by decreases in the number of TUNEL-positive cells and Bax expression as well as increase in Bcl-2 expression. NaHS upregulated the expression of Sirt1 in the hippocampus of CRS-exposed rats. Furthermore, inhibited Sirt1 by Sirtinol reversed the protective effects of NaHS against CRS-produced cognitive dysfunction and oxidative stress, ER stress as well as apoptosis in hippocampus. Together, these results suggest that H_2_S meliorates CRS-induced hippocampal damage and cognitive impairment by upregulation of hippocampal Sirt1.

## INTRODUCTION

Mental tension, emergency incident or other stimulating factors can make the body produce stress reaction. Within the scope of physiological stress response is benefit for the body [1]. However, When people are in long-term chronic stress situation, their physical and mental health are adversely influenced [2]. Chronic restrain stress (CRS), as a non-invasive stimulation, is able to better simulate a living state of uncontrollable congestion, setbacks in our daily life. It has been reported that CRS can impair hippocampal-dependent spatial learning and memory [3], which may be related to stress-caused oxidative damage in the hippocampal neurons and changes in synaptic structure. CRS is correlated with many neurodegenerative diseases, such as Alzheimer diseases [4]. Therefore, further investigation of the potential therapeutic approaches for treatment of CRS-induced hippocampal damage and cognitive impairment will provide new opportunities for improving human health.

Hydrogen sulfide (H_2_S), the third gasotransmitter along with nitric oxide (NO) and carbon monoxide (CO) [5], plays potent protective effects in the central nervous system [6, 7]. Accumulating evidences demonstrate that H_2_S facilitates the induction of hippocampal long term potentiation (LTP) [8, 9] and attenuates hepatic I/R- or β-amyloid-induced impairment in spatial learning and memory [10-13]. Moreover, our recent study certified that disturbance of endogenous H_2_S generation in hippocampus is involved in deficits in learning and memory [14, 15] and that H_2_S antagonizes formalydehyde-exerted deficits in cognitive function [16]. Thus, the present work was to explore whether H_2_S ameliorates CRS-induced cognitive impairment.

Sirt1 (silent information regulator1), as an nicotinamide-adenine dinucleotide (NAD^+^)-dependent histone deacetylase [17], is originally found to increase DNA stability and extend lifespan in yeast and higher organisms [18, 19], including mammals [20]. Accumulating evidence suggests that Sirt1 plays a critical role in CNS via reglulating diverse intracellular activity [21-23]. Growing evidence demonstrates that Sirt1 is expressed in neurons of the hippocampus and modulates cognitive performance and memory formation [24, 25]. Meanwhile, Sirt1 deficiency impaired cognitive abilities, including immediate memory and spatial learning [25]. Furthermore, our recent study testified the upregulatory role of H_2_S in hippocampal Sirt1 [26]. Therefore, we will explore whether the protection of H_2_S against CRS-induced cognitive impairment is also via upreglulating hippocampal Sirt1.

Based on hippocampus is a key structure closely related with learning and memory, we still explored the effects of H_2_S against CRS-induced hippocampal damage. The present work identified that H_2_S not only attenuated the cognitive impairment but also inhibited the hippocampal damage in the CRS-exposed rats. We also demonstrated that H_2_S significantly upregulated SIRT1 expression in the hippocampus of CRS-exposed rats. Furthermore, Sirtinol, a Sirt1 inhibitor, reversed the protective effects of H_2_S against CRS-induced hippocampal damage and cognitive impairment. Taken together, we identified a critical role of H_2_S in the protection against CRS-induced hippocampal damage and cognitive impairment, as a result of upregulation of hippocampal Sirt1.

## RESULTS

### H_2_S ameliorates the cognitive impairment in CRS-exposed rats

To investigate the ameliorating role of H_2_S in CRS-induced cognitive impairment in rats, we explored the effect of NaHS (a donor of H_2_S, 30 and 100 μmol/kg/d, i. p. for 28 d) on the cognitive function of CRS (6 h/d for 28 d)-exposed rats by Y-maze test, Novel object recognition test, and Morris water maze test.

**Figure 1:**
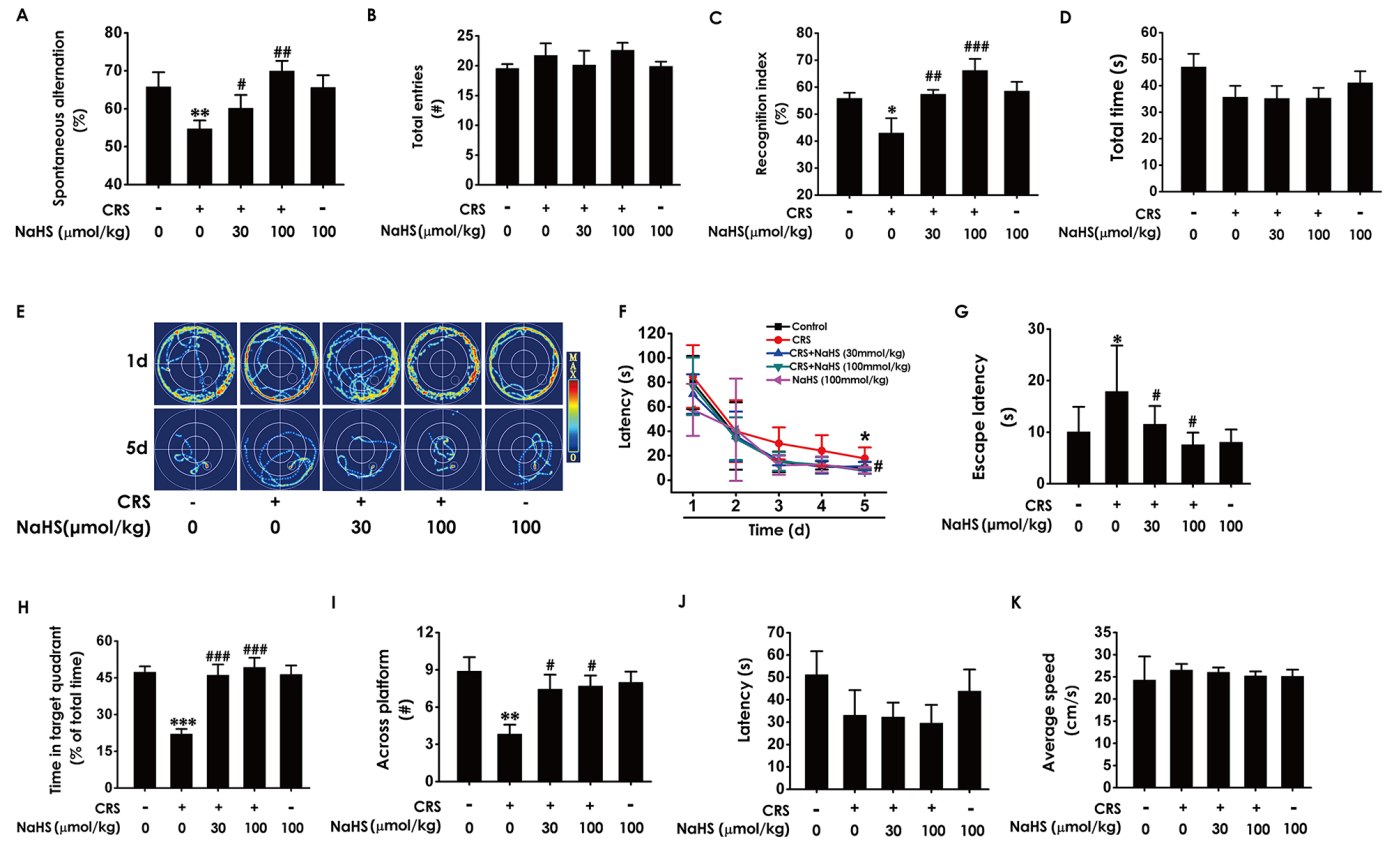
Effects of H_2_S on CRS-induced learning and memory impairment in rats After cotreated with NaHS (30 and 100 μmol/kg/d, i.p.) and CRS (6 h/d) for 4 w, the rats were tested in the Y-maze test **(A-B)**, the novel object recognition test **(C-D)**, and the Morris Water Maze **(E-K)**. (A-B) The total arm entries (A) and the alternation performance (B) were recorded; (C-D) the discrimination index (C) and the total object exploration time of rats (D) in test period was recorded. (E-K) The swimming tracks of rats searching for the underwater platform at the 1st and 5th training days (E) and the latency traveled to find the platform during five days (F) in the acquisition phase was recorded; the number of times that the rats crossed the platform (H) and the percentage of time in target quadrant (I) in probe trial were recorded; the latency to reach the platform (J) and the average speed of rats (K) in the visible platform test were recorded. Values were presented as mean ± S.E.M. (n=8-10). ^*^P < 0.05, ^**^P < 0.01,^***^P < 0.001, *vs* control group; ^#^*P* < 0.05, ^*##*^*P* < 0.01,^###^*P* < 0.001, *vs* CRS-treated alone group.

In Y-maze test, NaHS prominently increased the alternation performance in CRS-exposed rats (Figure 1A) and there was no significant difference in total arm entries among the six groups (Figure 1B). In novel object recognition test, NaHS significantly increased the recognition index in CRS-exposed rats (Figure 1C) and there was no difference in the total amounts of exploration time among the six groups (Figure 1D). These findings revealed that H_2_S attenuated CRS-induced impairment in cognition.

We further explored the beneficial effect of H_2_S on cognitive function of CRS-exposed rats using the Morris water maze (MWM) test. In the 5^th^ of acquisition phase, NaHS simplified the swimming routes (Figure 1E) and concurrently shortened the escape latency to the hidden platform (Figure 1F and 1G) in CRS-exposed rats, indicating that NaHS improved the spatial learning of CRS rats. In the probe trial, NaHS increased the times of crossing platform (Figure 1H) and proportionality of swimming time in target quadrant (Figure 1I) in CRS-exposed rats, indicating that NaHS improved the spatial memory of CRS rats. In the visible platform test, all rats among five groups showed similar latencies to the platform and average speed (Figure 1J and 1K), indicating their normal visual perception and swimming capability. Together, these data suggested that H_2_S meliorates CRS-induced cognitive impairment.

### H_2_S protects against CRS-generated hippocampal oxidative stress

**Figure 2:**
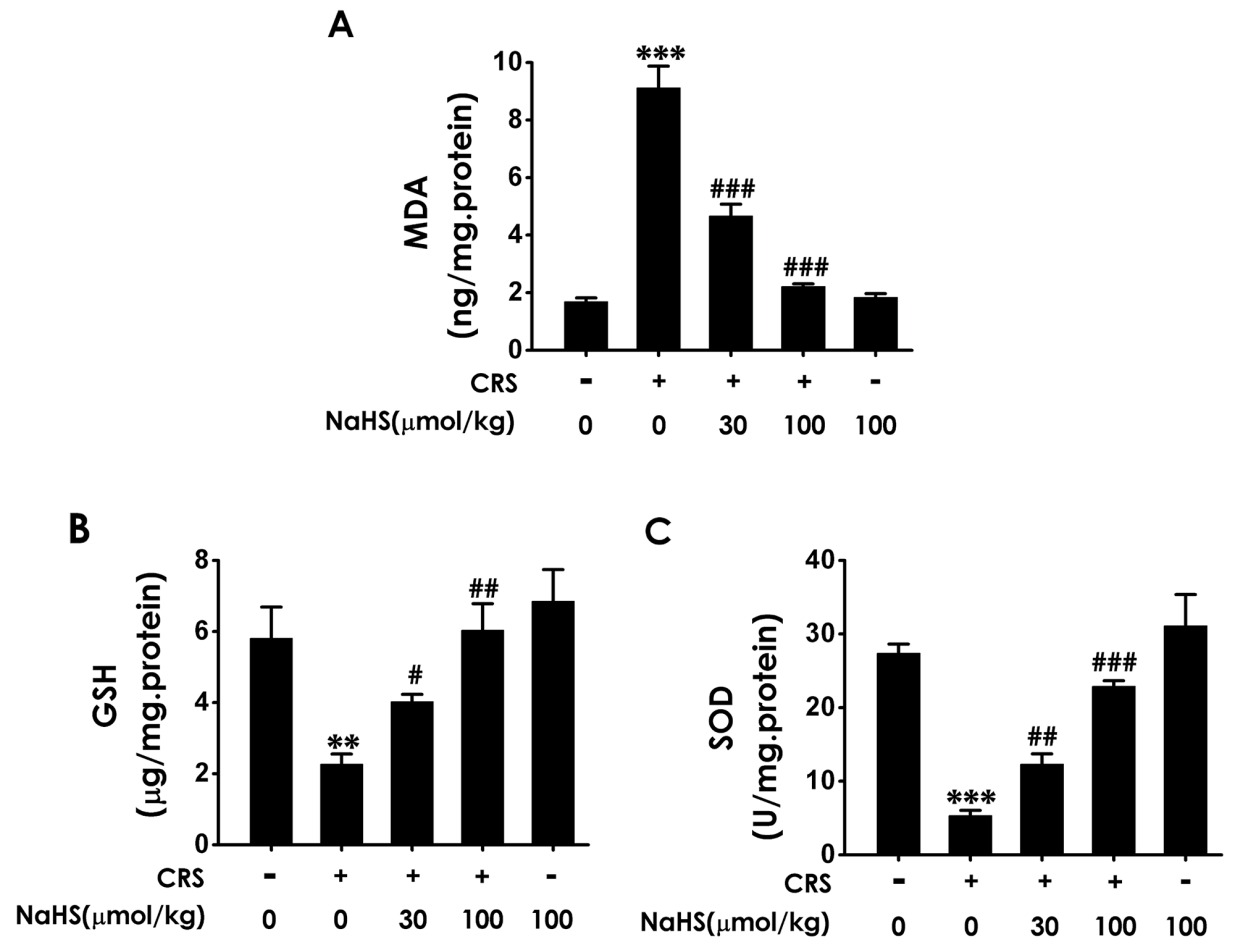
Effect of H_2_S on CRS-exerted hippocampal oxidative stress in rats 1 day after behavior tests, the hippocampus of rats were collected. The level of MDA **(A)** and the content of GSH **(B)** in hippocampus were detected by ELISA kit. The activity of SOD **(C)** was measured by the NBT assay kit. Values are the means ± SEM (n=3). ^**^P < 0.01,^***^P < 0.001, vs control; ^#^*P* < 0.05, ^##^*P* < 0.01, ^###^*P* < 0.001, vs CRS-treated alone group.

MDA is the marker of lipid peroxidation to indicate the oxidative stress level. Treatment with NaHS significantly reduced the levels of MDA (Figure 2A) in the hippocampus of CRS-exposed rats. We also examined the effects of H_2_S on the SOD activity and GSH level in the hippocampus of CRS-treated rats. NaHS (30 or 100 μmol/kg/d, i.p.) increased the SOD activity and GSH level in the hippocampus of CRS-treated rats (Figure 2B and 2C). These results support the protective action of H_2_S against CRS-induced hippocampal oxidative stress.

### H_2_S prevents the hippocampal ER stress triggered by the exposure of CRS

**Figure 3:**
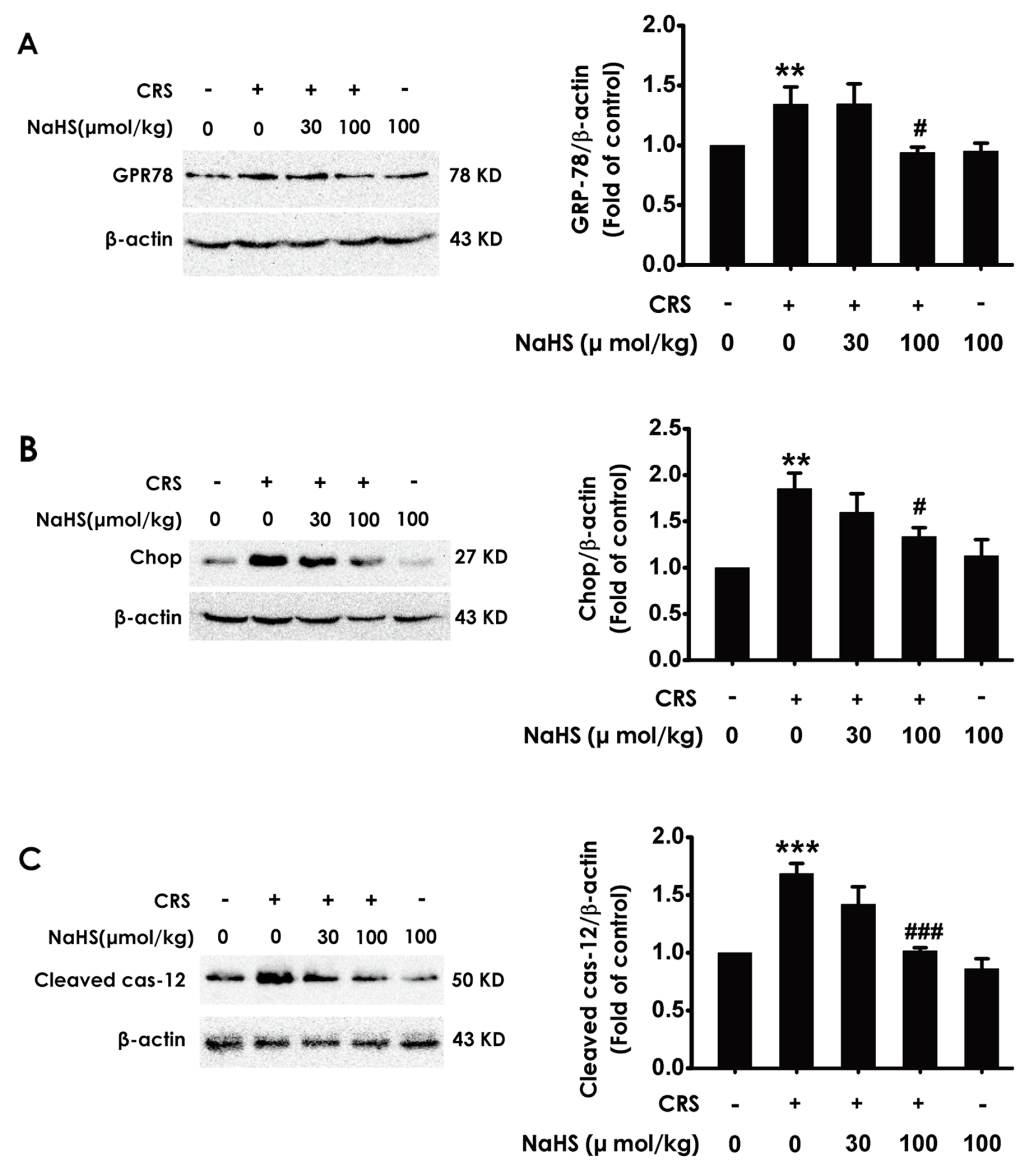
Effect of H_2_S on CRS-induced hippocampal ER stress in rats 1 day after behavior tests, the hippocampus of rats were collected. The expression of CPR78 **(A)**, CHOP **(B)**, and cleaved caspase-12 **(C)** were detected by western blotting. Values are the means ± SEM (n=3). ^**^P < 0.01, ^***^P < 0.001, vs control; ^#^*P* < 0.05,^###^*P* < 0.001, vs CRS-treated alone group.

To demonstrate whether H_2_S inhibits chronic-restrain-stress-induced hippocampal ER stress, we also investigated the effect of H_2_S on the expressions of CPR78, Chop, and cleaved caspase-12 in the hippocampus of CRS-treated rats. NaHS decreased the expressions of CPR78 (Figure 3A), Chop (Figure 3B), and cleaved caspase-12 (Figure 3C) in the hippocampus of CRS-exposed rats, which indicated the protective role of H_2_S against CRS-exerted hippocampal ER stress.

### H_2_S attenuates the hippocampal apoptosis in CRS-exposed rats

**Figure 4:**
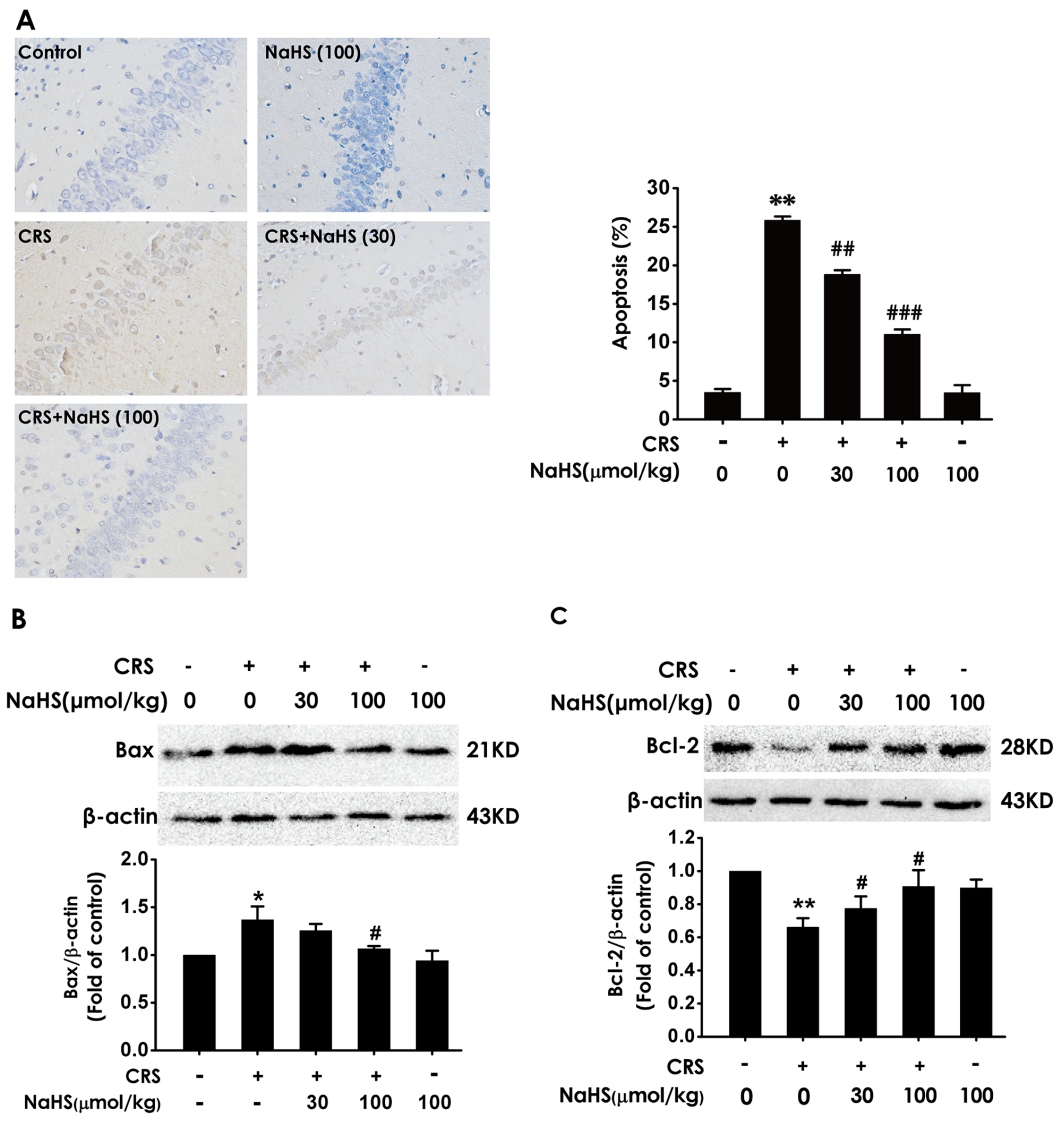
Effect of H_2_S on CRS-induced hippocampal apoptosis in rats 1 day after behavior tests, the hippocampus of rats were collected. **(A)** The level of apoptosis was detected by tunel staining (Left, magnification x400). (B and C) The apoptotic-associated proteins Bax **(B)** and Bcl-2 **(C)** were measured by western blotting. Values are expressed as the mean ±S.E.M. (n=3 per group). ^*^P < 0.05, ^**^P < 0.01, *vs* control group; ^#^*P* < 0.05, ^*##*^*P* < 0.01,^###^*P* < 0.001, *vs* CRS-treated alone group.

We detect the apoptotic cells in the hippocampus slices by TUNEL staining and the apoptosis-associated protein Bax and Bcl2 to confirm the protection of H_2_S against CRS-induced apoptosis. After treatment with NaHS, the TUNEL-positive neurons (Figure 4A) and the level of Bax (Figure 4B) in the hippocampus of CRS-exposed rats were significantly decreased, while the level of Bcl2 in the hippocampus of CRS-exposed rats was significantly increased (Figure 4C), which indicated the protective action of H_2_S on CRS-induced apoptosis.

### H_2_S upregulates hippocampal Sirt1 expression in CRS-exposed rats

**Figure 5:**
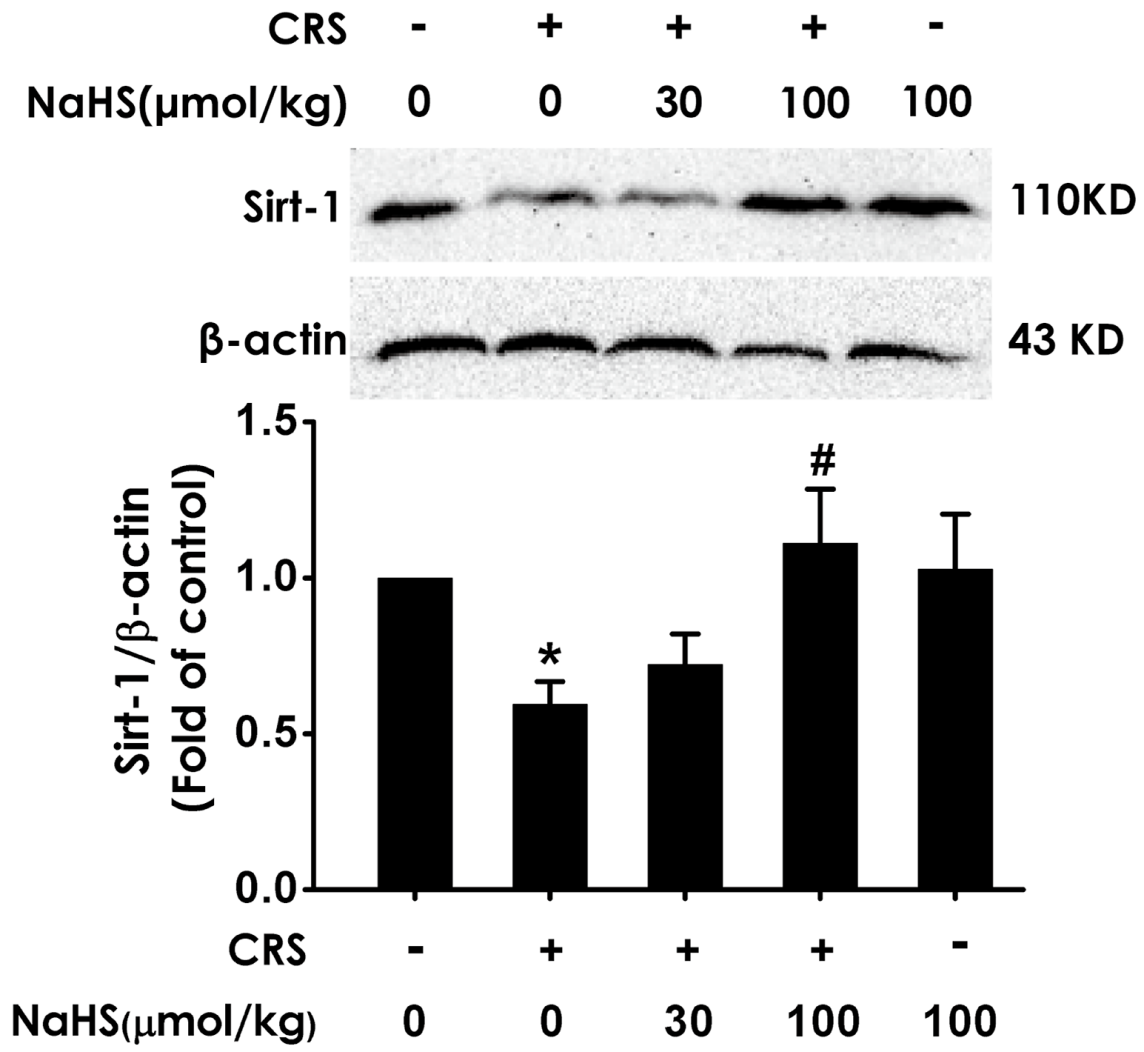
Effect of H_2_S on the level of hippocampal Sirt1 in the CRS-exposed rats 1 day after behavior tests, the hippocampus of rats were collected. Sirt1 was measured by western blotting. Values are the means ± SEM (n=3). ^*^P < 0.05, vs control; ^*#*^*P* < 0.05, vs CRS-treated alone group.

To investigate whether Sirt1 mediates the protective effect of H_2_S against CRS-induced hippocampal damage and cognitive impairment, we first explored the effect of H_2_S on the expression of Sirt1 protein in CRS-exposed rats. After 4-w exposure of CRS, Sirt1 level in the hippocampus of rats was markedly decreased (Figure 5A). However, after cotreated with NaHS (30, 100 μmol/kg/d, i.p., for 28 d), the expression of Sirt1 in CRS-exposed rats was markedly increased (Figure 5A). These data support that H_2_S restores the expression of hippocampal Sirt1 in CRS-exposed rats.

### Inhibition of Sirt1 blocks the inhibitory role of H_2_S in CRS-exerted cognitive deficits

**Figure 6:**
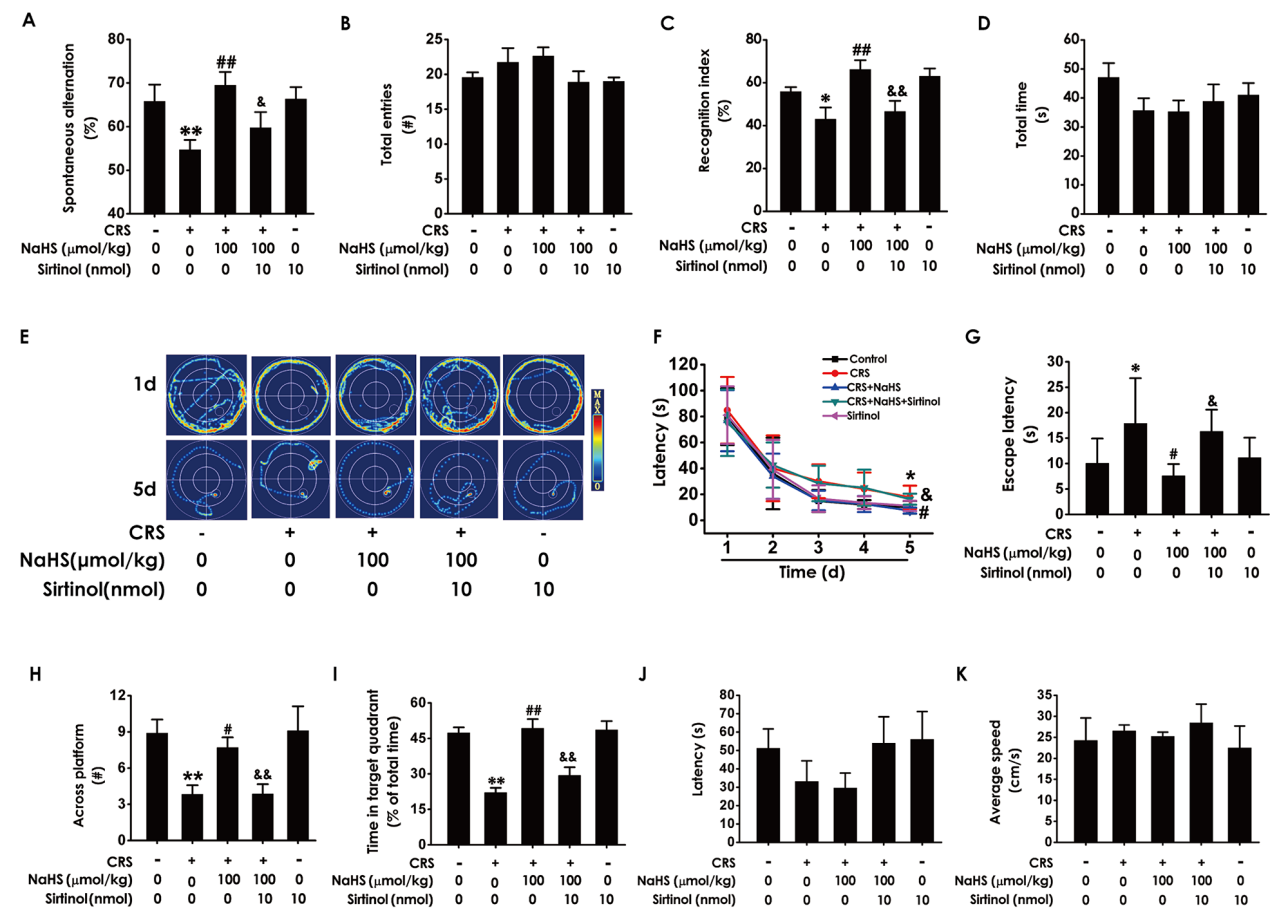
Effects of Sirtinol on H_2_S-meliorated the function of cognition in CRS-treated rats Rats were cotreated with NaHS (100μmol/kg/d, i.p.) and CRS (4h) for 4 w and injected with Sirtinol ((10 nmol/d ×1w, i.c.v.) at the last week simultaneously. The rats were tested in the Y-maze test **(A-B)**, the novel object recognition test **(C-D)**, and the Morris Water Maze **(E-K).** Values are the mean ± S.E.M. (n=8-10). (A-B), the total arm entries (A) and the alternation performance (B) were recorded; (C-D), the discrimination index (C) and the total object exploration time of rats (D) in test period was recorded. (E-K), the swimming tracks of rats searching for the underwater platform at the 1st and 5th training days (E) and the latency traveled to find the platform during five days (F) in the acquisition phase was recorded; the number of times that the rats crossed the platform (H) and the percentage of time in target quadrant (I) in probe trial were recorded; the latency to reach the platform (J) and the average speed of rats (K) in the visible platform test were recorded. ^*^P < 0.05, ^**^P < 0.01, vs control group; ^#^*P* < 0.05, ^##^*P* < 0.01, vs CRS-treated alone group; ^&^*P* < 0.05, ^&&^*P* < 0.01, vs cotreated with CRS and NaHS (100 μmol/kg/d, i.p.) group.

To confirm the mediatory role of Sirt1 in the inhibitory role of H_2_S in CRS-induced cognitive deficits, we further explored whether Sirtinol (10nmol ×1w, i.c.v.), a specific Sirt1 inhibitor, reverses the protection of H_2_S against cognitive dysfunction in CRS-exposed rats. In the Y-maze test, Sirtinol (10 nmol ×1w, i.c.v.) eliminated the protection of NaHS (100 μmol/kg/d, i.p.) against CRS-induced decreases in the alternation performance, while treatment with Sirtinol (10 nmol ×1w, i.c.v.) alone did not affect the alternation performance in control rats (Figure 6A). All of five groups have no significant difference in arm entries (Figure 6B). In the Novel Object Recognition Test, Sirtinol (10nmol ×1w, i.c.v.) induced decrease in the recognition index of cotreatment with NaHS (100 μmol/kg, i.p.) and CRS rats (Figure 6C), whereas there were no significant difference in the total amounts of exploration time among five groups (Figure 6D). These findings revealed that inhibition of Sirt1 blocked the protective effect of H_2_S on CRS-exerted cognitive deficits.

We further tested whether Sirtinol reverses the beneficial role of H_2_S in the spatial learning and memory ability of CRS-exposed rats using Morris water maze (MWM) test. In the 5^th^ of acquisition phase, Sirtinol reversed the simplified effect of H_2_S on the swimming routes in CRS-exposed rats (Figure 6E). Concurrently, Sirtinol eliminated the meliorated effect of H_2_S on the escape latency to the platform in CRS-exposed rats (Figure 6F and 6G). In probe trial test, Sirtinol decreased the times of crossing platform (Figure 6H) and proportionality of swimming time in target quadrant (Figure 6I). In the visible platform test, all rats among five groups showed similar latencies to the platform and average speed, indicating their normal visual perception and swimming capability (Figure 6J and Figure 6K). Together, these data suggested that Sirtinol eliminates the protective effect of H_2_S on CRS-exerted cognitive deficits.

### Sirtinol blocked the antioxidant effect of H_2_S on CRS-exerted hippocampal oxidative stress

**Figure 7:**
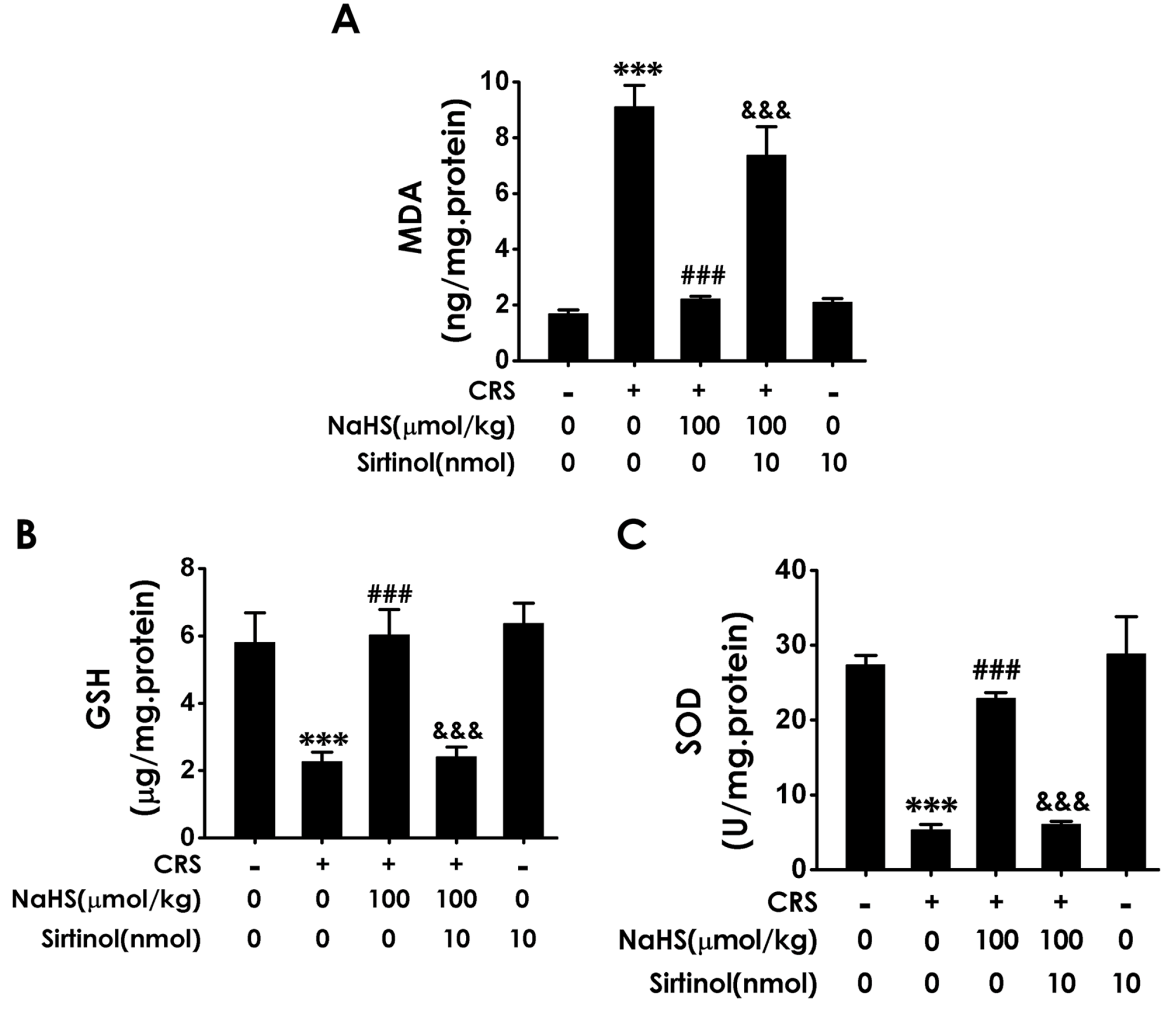
Effect of Sirtinol on H_2_S-meliorated hippocampal oxidative stress in CRS-treated rats Rats were cotreated with NaHS (100 μmol/kg/d, i.p.) and CRS (6 h/d) for 4 w and injected with Sirtinol (10 nmol/d×1w, i.c.v.) at the last week simultaneously. The level of MDA **(A)** and GSH **(B)** were detected by ELISA kit. The activity of SOD **(C)** was measured by the NBT assay kit. Values are the means ± SEM (n=3). ^***^P < 0.001, vs control; ^###^*P* < 0.001, vs CRS-treated alone group; ^&&&^*P* < 0.001, vs cotreated with CRS and NaHS (100 μmol/kg/d, i.p.) group.

To confirm the involvement of Sirt1 in the protection of H_2_S against CRS-induced hippocampal damage, we first explored whether Sirtinol, a specific Sirt1 inhibitor, reverses the protective role of H_2_S against hippocampal oxidative stress in CRS-exposed rats. As shown in Figure 7, Sirtinol (10 nmol ×1w, i.c.v.) reversed the protection of NaHS (100 μmol/kg/d, i.p.) against CRS-induced increase in hippocampal MDA level (Figure 7A) as well as decreases in hippocampal GSH content (Figure 7B) and SOD activity (Figure 7C). Together, these data indicated that Sirtinol blocks the protection of H_2_S against CRS-induced hippocampal oxidative stress in rats.

### Sirtinol reverses the meliorating effect of H_2_S on CRS-exerted hippocampal ER stress

**Figure 8:**
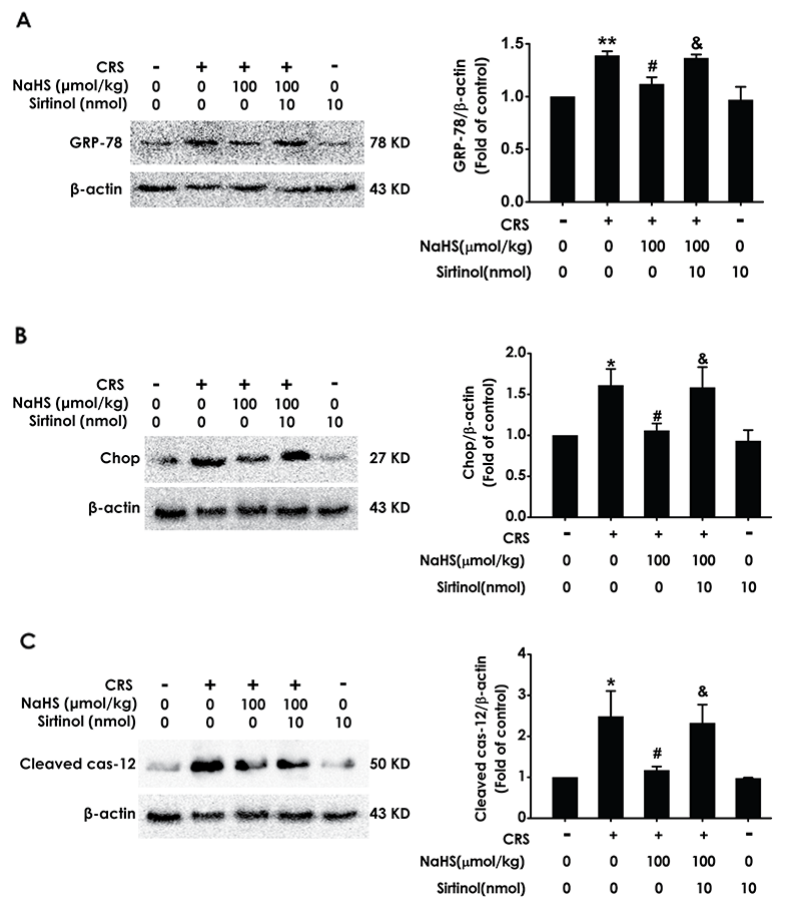
Effect of Sirtinol on H_2_S-meliorated hippocampal ER stress in CRS-exposed rats Rats were cotreated with NaHS (100 μmol/kg/d, i.p.) and CRS (6 h/d) for 4 w and injected with Sirtinol (10 nmol/d ×1w, i.c.v.) at the last week simultaneously. The expression of CPR78 **(A)**, CHOP **(B)**, and cleaved caspase-12 **(C)** were detected by western blotting. Values are the means ± SEM (n=3). ^*^P < 0.05, ^**^P < 0.01, vs control; ^#^*P* < 0.05, vs CRS-treated alone group; ^&^*P* < 0.05, vs cotreated with CRS and NaHS (100 μmol/kg/d, i.p.) group.

Next, we determine whether Sirtinol reverses the protective role of H_2_S on CRS-induced ER stress. As shown in Figure 8, Sirtinol (10 nmol ×1 w, i.c.v.) increased in the expressions of GPR78 (Figure 8A), Chop (Figure 8B), and cleaved caspase-12 (Figure 8C) protein in the hippocampus of cotreatment with NaHS and CRS rats, which indicated that Sirtinol reverses the protective action of H_2_S on CRS-induced in hippocampal ER stress.

### Sirtinol antagonizes H_2_S-ameliorated hippocampal apoptosis in CRS- exposed rats

**Figure 9:**
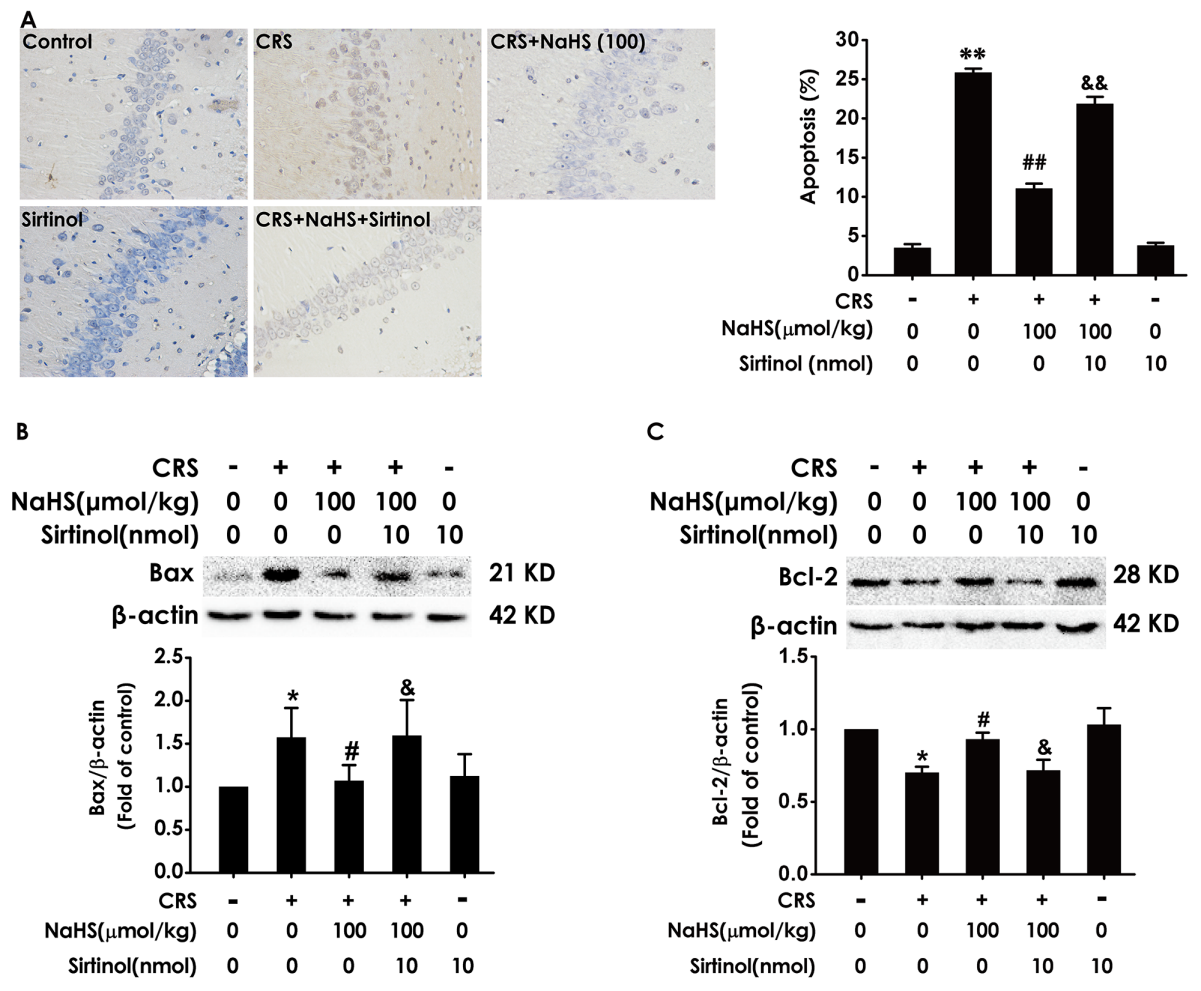
Effect of Sirtinol on H_2_S-meliorated hippocampal apoptosis in CRS-treated rats Rats were cotreated with NaHS (100 μmol/kg/d, i.p.) and CRS (6 h/d) for 4 w and injected with Sirtinol (10 nmol/d×1w, i.c.v.) at the last week simultaneously **(A)** The level of apoptosis was detected by tunel staining (Left, magnification x400). (B and C) The apoptotic-associated proteins Bax **(B)** and Bcl-2 **(C)** were measured by western blotting. Values are the mean ± S.E.M. (n=3). ^*^P < 0.05, ^**^P < 0.01, vs control group; ^*#*^*P* < 0.05, ^*##*^*P* < 0.01, vs CRS-treated alone group; ^&^*P* < 0.05, ^&&^*P* < 0.01, vs cotreated with NaHS and CRS (100 μmol/kg/d, i.p.) treated group.

We also explored whether Sirtinol blocks the protective role of H_2_S in CRS-induced hippocampal apoptosis. After treatment with Sirtinol (10 nmol, i.c.v.), the amount of TUNEL-positive neurons (Figure 9A) and the level of Bax (Figure 9B) in the hippocampus of cotreatment with NaHS and CRS-exposed rats were significantly increased, while the level of Bcl2 in the hippocampus of cotreatment with NaHS and CRS-exposed rats was significantly decreased (Figure 9C), indicated that Sirtinol abrogates the protective action of H_2_S on CRS-induced apoptosis.

## DISCUSSION

H_2_S acts as an important modulatory role in learning and memory functions [27]. Numerous studies supports that chronic restrain stress (CRS) impairs hippocampal-dependent spatial learning and memory [28-30]. In this study, we investigated the protective effects of H_2_S against CRS-induced hippocampal damage and cognitive impairment and further dissected its mechanism. We found that H_2_S attenuated hippocampal damage and cognitive impairment, coupled with increasing the expression of Sirt1 in the hippocampus of CRS-exposed rats. In addition, this protective effect of H_2_S against hippocampal damage and cognitive impairment was abolished by Sirtinol, an inhibitor of Sirt1. These discoveries together suggested that H_2_S attenuates CRS-induced hippocampal damage and cognitive impairment by upreglulation of hippocampal Sirt1.

Chronic restrain stress in our daily life is a potential risk factor for human health [31]. Extensive evidence from animal and human studies indicates that CRS induces a deficit in cognitive function [29, 32]. H_2_S, a gaseous signaling molecule, scavenges ROS and protects neurons against oxygen stress damage, neurotoxicity, and cell apoptosis [33, 34]. Furthermore, H_2_S plays an important role in regulation of learning and memory [10]. To investigate the beneficial effect of H_2_S on CRS-induced deficits in learning and memory, rats were cotreated with NaHS and CRS for 28 d, and the functions of learning and memory of rats were tested with Y-maze test, Novel object recognition test, and Morris water maze test. In Y-maze test, we showed that the alternation performance in CRS -treated alone rats was increased by treatment with NaHS, which indicated that H_2_S restores CRS-induced impairments in learning and memory. In the Novel object recognition test, we showed that the discrimination index in CRS-treated alone rats was significantly increased by treatment with NaHS, which also indicates the harmful role of CRS in learning and memory can be reversed by H_2_S. In MWM test, we showed that treatment with NaHS decreases the escape latency in hidden-platform acquisition training and increases the crossing platform times and the percentage of time in the target quadrant in the probe trail in CRS-exposed rats, which indicated that H_2_S reverses the impairment in spatial learning and memory induced by CRS. Taken together, our present work demonstrates that H_2_S prevents CRS-induced cognitive impairment in rats.

Hippocampus is one of the limbic structures involved in cognition [35]. Furthermore, hippocampus is also one of the most vulnerable structures to stress condition in the brain [36, 37] and its damage played a key role in CRS-induced cognitive impairment [38, 39]. To explore the potential beneficial effects of H_2_S on CRS-induced impairments in hippocampus, we detected the levels of hippocampal oxidative stress, ER stress and apoptosis. Our present work showed that H_2_S reversed CRS-exerted increase in MAD level, decrease in SOD activity, and decline in GSH level, which indicated that H_2_S protects against CRS-generated hippocampal oxidative stress. Meanwhile, H_2_S inhibited the expressions of ER stress-related proteins, GPR78, Chop and cleaved caspase-12 level, in the hippocampus of CRS-exposed rats, which indicated H_2_S protects CRS-generated hippocampal ER stress. Moreover, we also indicated the protection of H_2_S against CRS-generated hippocampal apoptosis, as evidenced by decreases in the number of Tunel positive cells and the expression of Bax as well as increase in the expression of Bcl2 in the hippocampus of CRS-exposed rats. In summary, these data indicated the alleviating role of H_2_S in CRS-elicited hippocampal damage and suggested that the protective effect of H_2_S against hippocampal damage is involved in its inhibitory role in CRS-induced cognitive impairment.

Sirt1 is a member of sirtuin famlily encoding NAD^+^-dependent deacetylases [40]. Numerous studies have documented that Sirt1 mediates the neuroprotective effects of resveratrol in neurodegenerative disease, such as Huntington disease [41]. Interestingly, our previous work demonstrated that H_2_S increases the expression of Sirt1 protein in hippocampus of rats [26]. Sirt1 also reglulates many cellular pathways involved in cellular stress responses, apoptosis, and axonal degeneration [42-44]. Our present work showed that CRS caused decreases in the expression of Sirt1 in hippocampus and H_2_S markedly increased the expression of Sirt1 in the hippocampus of CRS-exposed rats. We further found that Sirtinol, the Sirt1 inhibitor, reversed the protection of H_2_S against CRS-elicited oxdative stress, as evidenced by increase in MAD level and decreases in SOD activity and GSH level, ER stress, as evidenced by upregulation of GPR78, Chop and cleaved caspase-12 level, and apoptosis, as evidenced by increases in the number of Tunel positive cells and the expression of Bax as well as decrease in the expression of Bcl2 in the hippocampus of cotreatment with NaHS and CRS rats. Furthermore, Sirtinol reversed the protective effect of H_2_S against CRS-induced cognitive dysfunction reflected by Y-maze test, Novel object recognition and Morris water maze. It has been reported that Sirt1 is indispensable for cognitive function [25]. Taken together, our data suggested that the upregulation of hippocampal Sirt1 mediates the protective role of H_2_S in CRS-induced hippocampal damage and cognitive dysfunction.

In conclusion, our results revealed that H_2_S suppressed CRS-evoked hippocampal damage and cognitive impairment, which is mediated by the upreglulation of hippocampal Sirt1 in CRS-exposed rats. Our findings uncover the pivotal role of H_2_S in CRS-induced hippocampal damage and cognitive impairment and identify H_2_S as a potential therapeutic strategy for the pathogenesis of chronic stress.

## MATERIALS AND METHODS

### Reagents

Sodium hydrosulfide (NaHS, a donor of H_2_S) was purchased from Sigma (Sigma, St. Louis, MO, USA). Pelltobarbitalum Natricum was obtained from Germany and the Sirt1-antibody was purchase from American Abcam. The primary antibodies of CPR78, CHOP, cleaved caspase-12, Bax and Bcl2 were bought from cell signaling technolgy. The malondialdehyde (MDA) assay kit was bought from Uscn Life Science Inc. (Wuhan, Hubei, China). The glutathione (GSH) enzyme-linked immunosorbent assay (ELISA) kit was purchased from Bio-Swamp Life Science. Total SOD assay kit and Bicinchoninic Acid (BCA) Protein Assay Kit were obtained from Beyotime Institute of Biotechnology (Shanghai, China). Specific monoclonal antibody for detecting Sirtinol was purchased from Santa. Bicinchoninic Acid (BCA) Protein Assay Kit was obtained from Beyotime Institute of Biotechnology (Shanghai, China).

### Animals

Adult male Sprague-Dawley (SD) rats (200–220 g) were purchased from the Hunan SJA Laboratory Animal Center (Changsha, Hunan, China). Rats were housed individually and given free access to food and water under a normal 12 h light/dark schedule (lights on at 07:00 a.m.). Room temperature was maintained at 22 ± 1 °C and relative humidity of 55% ± 5%. All rats were allowed 7 days to adapt to the housing conditions before the beginning of the experiments. All the experiments were conducted in accordance with the National Institutes of Health Guide for the Care and Use of Laboratory Animals and were approved by the Animal Use and Protection Committee of University of South China.

### Drug treatments and experimental schedule

**Figure 10:**
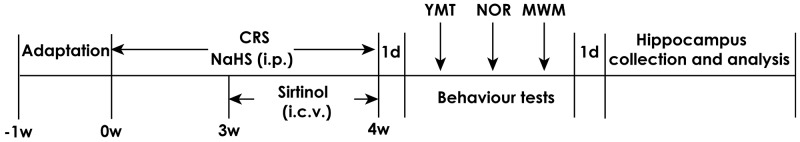
Schematic diagram of the experimental schedule CRS, chronic restraint stress; YMT, Y maze test; NOR, novel object recognition; MWM, morris water maze; i.p., intraperitoneal injection; i.c.v., intracerebroventricular injection.

All rats were randomly divided into seven groups: Control group, rats were general treated without stress for 4 w and injected with PBS (i.p.) for 4 w and artificial cerebrospinal fluid (ACSF, i.c.v.) in the last 1 w; CRS group, CRS rats were bound in a well-ventilated 50 ml volume of stainless steel tube in 6 h (09:00-15:00) for 4 w and simultaneously received 4 w of PBS (i.p.); CRS +NaHS (30 μmol/kg or 100 μmol/kg) group, CRS rats were exposed to stress and coinjected with 30 μmol/kg or 100 μmol/kg NaHS for 4 w; CRS + NaHS (100 μmol/kg) + Sirtinol (10 nm, i.c.v.) group, CRS rats were exposed to stress and coinjected with 100 μmol/kg/d NaHS (i.p.) for 4 w and 10 nm Sirtinol (i.c.v.) for the last week of the four-week CRS procedure; NaHS (100 μmol/kg) alone group and Sirtinol alone group, rats were conventionally treated for 1 w and injected with NaHS (100 μmol/kg, 4 w, i.p.), and Sirtinol (10 nmol, 7 d, i.c.v.). Sirtinol (5mg) was dissolved in 1269 μL of dimethysulfoxide (DMSO) to 10 nmol/μL of a mother liquid. And then 20 μL of mother liquor was diluted in 20μL of DMSO to 5 nmol/μL of working solution. All behavior tests were performed after 24 h of the last injection. On the next day of behavior tests, all rats were killed, and hippocampus were rapidly collected and stored at -80 °C for analysis (Figure 10)

### Intracerebroventricular injection

After animals were deeply anesthetized using 1% sodium pentobarbital (0.04 ml/kg, i.p.), animals were placed in a stereotaxic frame for operation. The area around the incision was clipped by sterile surgical scissors. Sirtinol (10 nmol) was injected unilaterally into the ventricle with an injection rate of 0.75 μL/min using a 10-μL Hamilton syringe using the following coordinates: AP: 1.0 mm, R or L: 1.5 mm. To ensure drug had been completely delivered, the needle should be slowly pulled out halfway and kept in position for extra 2 minutes before moving out. All rats were injected subcutaneously with penicillin during 3 consecutive days after operation.

### Y-maze test

Y-maze test is mainly used for functional assessment of animal learning and memory. In order to eliminate the effects of visual and olfactory cues on experimental result, Y-maze must be clear, symmetry, and three arms inside Y maze are coated with black paint which avoids seeing the other arm outside the maze. It contains three arms of 120° angle which were designated as arm A, B and C. Each arm is 50 cm x 18 cm x 35 cm. The experimental rats were placed in one arm and let it freely inquiry in three arm within 8 min, and record the order and the times of mouse enter into each arm. When four legs of rats all entered into the dividing line of one arm, this situation was thought as rats enter into one arm. Rats entered into three different arms in turn, which was considered as a correct alternating sequence (for example, ABC ACB BCA, etc.). Rats repeatedly entered into one arm in three consecutive chances, which was defined as an incorrect alternating sequence (namely the ABA, BCB, CAC, etc.). In order to eliminate the effects of smell on the experiment, all arms and their bottoms should wiped using alcohol. The spontaneous alternation (= (total correct alternating sequence/total alternating sequence) × 100%) was recorded to evaluate the spatial orientation learning ability of rats and testing their activity.

### Novel object recognition test

Novel object recognition test is used to evaluate the rat hippocampal related cognitive function. It includes three phases: adaptive, training and testing phase. In adaptive phase, each rat was placed in empty behavioral box (50.0 cm × 50.0 cm ×60 cm) without any items (5 min each day for 2 consecutive days). In training phase, two cylinderical objects (A and B) which contain certain weight were placed in the behavioral box, and rats were allowed respectively to explore in the behavioral box for 5 minutes, and recorded the time of exploring each object with A stopwatch. After the training period, each mouse has 60 minutes interval and then entered the test period. In the test period, two different objects (A, the previous cylinderical object and C, a cubical object) were placed in the behavior box, and rats were allowed respectively to explore in the behavioral box for 5 minutes, and recorded the time of exploring each object. The whole experiment process was recorded by video. The behavioral box was scrubed using alcohol after each test. The location ofnovelobject between different groups was random. Mouse climb or chewing objects are not seen as exploratory behavior. Exploratory behavior is defined as smell, touch, and pay attention to the object directly, and rat‘s nose away from the object distance within 1 cm is considered as exploratory behavior. The distinguish index (= (novel object − familiar object)/(novel object + familiar object)) of rats was computed to for the measurement of cognitive function in rats after the whole experiment.

### Morris water maze test

Morris water maze (Morris water maze, MWM) test is widely used in the measurement of rodent space learning, memory and working memory [45, 46]. Rats after handling were trained to find a submerged platform in a water maze using four visual cues surrounding the pool to test their spatial learning followed by a probe trial to test their memory retention. Prior to the first training trial, rats were given a single habituation trial without the platform to assess any spatial bias and their basal swim speed. For training (day1-5), rats were randomly introduced to different start locations of the pool for each trial with the hidden platform maintained in the same quadrant (target quadrant). Swim path and latency to locate the platform was tracked and determined by an MT-200 Morris Image Motion System (Chengdu Technology and Market Corp). For probe test (day5), the platform was removed and the swimming in each quadrant and specifically the preference for the target quadrant was measured to evaluate spatial memory. Visual and sensorimotor skills were assessed with a visible platform placed at various locations after the probe test.

### Biochemical analysis for MDA, GSH and SOD

The hippocampus tissue was homogenized and then centrifuged at 12,000 g for 10 minutes. The supernatants were collected and total protein contents were detected using BCA Protein Assay. The generation of MDA and GSH were measured by ELISA kits. And the activity of SOD was measured by the NBT assay kits. Detail steps were according to the manufacturer’s instruction on the assay kits.

### Western-blot detection for Bax, Bcl2, CPR78, CHOP, cleaved caspase-12 and Sirt1 expression in hippocampus tissue

Collected supernatant of sample and assessed total protein concentration by BCA Protein Assay Kits. Then the protein was diluted by PBS to same concentration. Protein extract with an equivalent volume for each sample was run on sodium dodecyl sulfate-polyacrylamide gel electrophoresis. After that, the protein was transferred to PVDF membrane using wet transfer system and blocked with TBST (50 mmol/L Tris-HCl, pH 7.5,150 mmol/L NaCl, 0.1% Tween-20) at room temperature. 2 hours later, membranes were respectively incubated with primary antibodies against Bax, Bcl2, CPR78, CHOP, cleaved caspase-12 (1:1000), Sirt1and β-actin (1:2000) at 4 °C overnight. Next day, membranes were washed with TBST for 3 × 10 min and were incubated for 2 hours with anti-rabbit secondary antibody diluted 1:5000. Finally, wash membranes with TBST in the same way and detect the expression of protein by enhanced chemiluminescence system.

### Statistical analysis

Statistical analysis of all data was performed by SPSS 18.0 software. Data are displayed as the mean ± SEM. The significance of intergroup differences was evaluated by one-way ANOVA and LSD-t was applied to analysis of variance as well as multiple comparisons between groups. Differences were considered significance at *P* < 0.05.
